# The Well-Being of Siblings of Individuals with Autism

**DOI:** 10.1155/2013/417194

**Published:** 2013-04-30

**Authors:** Laura Green

**Affiliations:** College of Science, Engineering and Health, RMIT University, Melbourne, VIC 3083, Australia

## Abstract

*Objective*. The purpose of this review of the literature was to summarise studies regarding the psychosocial impact of growing up with a sibling with autism and to identify gaps in the related literature. *Methods*. Electronic databases were reviewed in order to critically appraise the 14 articles relevant to the topic. The search included a combination of the following key words: autism∗, quality of life, well-being, sibling∗, ASD, ASD sibling∗, family, adjust∗, psychological functioning. *Results*. The majority of studies involved mixed children and adolescent samples, leading to confounding results and an inability to draw accurate conclusions about these distinct life stages. Autism appears to contribute to unique environmental stressors for the typically developing sibling. When experienced in the context of additional demographic risk factors, these stressors can result in difficulties adjusting to the demands of a special-needs child. Despite some vulnerability to behavioural and emotional dysfunction in at-risk children, siblings have the potential to not only adjust but to thrive in the face of disability adversity. *Conclusion*. Growing up with a sibling with autism appears to manifest in both positive and negative outcomes for siblings, depending upon important demographical, family, and individual variables.

## 1. Introduction

The sibling bond is the marathon runner of human relationships; it is the longest lasting relationship, enduring from the birth of the youngest sibling to the death of the first to go. It is therefore reasonable to assume the importance of this relationship across the lifespan. 

 It has been established that the younger siblings of individuals with autism spectrum disorder (ASD) are at heightened risk of developmental problems in comparison to the general population. Some of these developmental difficulties include: social and cognitive deficiencies [1], and neurocognitive and behavioural delays, specifically executive function and repetitive behaviours [2]. The above examples of sibling research are commonly used to provide evidence for the genetic basis of ASD with elevated levels of autistic traits in siblings representing potential markers of a broader autism phenotype (BAP; [2]). The nurture side of the debate, however, is lacking considerable focus. How are these siblings and families impacted by the disorder in regard to their quality of life (QoL) and psychosocial outcomes? 

The American Psychiatric Association [3] describes the essential features of autistic disorder as “the presence of markedly abnormal or impaired development in social interactions and communication and a markedly restricted repertoire of activity and interests” (page 70). This is commonly referred to as the triad of autism diagnosis, representing delays or deficits in three essential categories: social interaction, communication, and restricted interests. Characteristic symptoms may include self-stimulation behaviours (e.g., hand flapping and rocking), self-injury behaviours (e.g., head banging, skin picking, hair pulling), lack of emotional and social reciprocity, and inflexible adherence to routines or rituals. In addition to common comorbidity with other medical, psychiatric, and developmental disorders, these ASD characteristics can be difficult for families to manage. Epilepsy, allergies, sleep disorders, conduct disorder, attention deficit/hyperactivity disorder (ADHD), gastro-intestinal disorders, anxiety disorders, intellectual disability and language disorders often cooccur with a diagnosis of ASD [4]. Furthermore, ASD requires substantial financial investment in therapy, medical treatment, and early intervention education; increased vigilance of the individual with autism; micromanagement of the family environment to ensure minimal sensory discomfort and maintenance of a highly predictable routine; often limited communication with the family member with autism; and many other family disturbances.

 Ensuring that an individual with ASD lives in an environment that caters for their complex needs requires a large commitment from the family. It is empirically intuitive to assume that quality of life (QoL) is impacted in these families. This has yet to be supported by research, with a lack of studies focusing on QoL outcomes in ASD families. 

A review of the sibling literature reveals both positive and negative effects of growing up with an individual with a disability. Siblings of children with autism have been shown to do more poorly on a number of outcome measures in comparison to siblings of other developmental disabilities and those with only typically developing siblings. This distinct population of siblings has been shown to exhibit higher levels of internalising and externalising disorders [5, 6], social and behavioural adjustment problems [7], hassles with sibling behaviour [8], and distressing emotions such as guilt [9].

In contrast, other researchers have found no difference in levels of adjustment between siblings of individuals with ASD and those with only typically developing siblings [10] or those with siblings with other developmental diagnoses [11]. Even more promising, the positive impacts of growing up with a sibling with ASD are becoming known. Siblings of individuals with autism have been reported to have less conflict in the sibling relationship [10], family resilience [12], and increased self-perceived competence [6, 13, 14]. They have also been shown to have a more positive opinion of the sibling relationship [15], positive psychosocial and emotional development conditional upon limited demographic risk factors [14], feelings of empathy for their sibling [16], and increased maturity [17].

 The autism sibling literature is yet to reach consensus; however, Macks and Reeve [14, page 1065] succinctly conclude that “having a sibling with autism may not be a risk factor in and of itself, and children with autism may even have a positive influence on the life of the nondisabled sibling. However, when multiple demographic risk factors are present, it becomes more difficult for the nondisabled sibling to deal with the child with autism, both emotionally and psychologically.” Macks and Reeve consider the contribution this potentially makes to the inconsistency currently plaguing sibling research. If those studies reporting positive influences included samples of demographically stable families, while those samples that exhibited negative impacts consisted of participants experiencing a high number of demographic risk factors, the true effects of growing up with a sibling with autism would be masked, justifying continued exploration of sibling outcome. 

In order to investigate this masking effect at surface level, the author conducted closer inspection of the studies listed above that showed no difference between siblings of individuals with ASD and comparison siblings. Demographic risk factors appeared to be stable in these samples. The majority of the siblings in the Pilowsky et al. [11] study were part of high-income families (25 out of 27 families). A large proportion of the sample in the Kaminsky and Dewey [10] study reported receiving high levels of social support. The majority of Bayat's resilient families used in his 2007 study were intact families with average incomes of 81,000 USD per year. Although the study included families with low income (23%), over 60 percent were in the high-income category. Had these samples included more demographically disadvantaged siblings, their outcome may have been poorer than comparison siblings. The low level of demographic risk may have contributed to the healthy levels of adjustment reported in these studies, a consideration that further supports Macks and Reeve's proposal. 

Recent literature reviews report mixed results and emphasise the large gaps in our current knowledge of sibling outcome, due in large part to inconsistency in methodology and a lack of rigorous sampling procedures [18, 19].

The purpose of this literature review was to summarise studies of the psychosocial impacts of growing up with a sibling with ASD, identify gaps in the literature, and propose future directions for sibling research in the autism spectrum field. Although it is recognised that genetic factors play an integral part in any research regarding ASD, the current paper focuses on psychosocial rather than biological components of sibling outcome.

In the context of the multitude of challenging behaviours exhibited by an individual with autism, it is intuitively appealing to predict that the well-being of siblings in these homes may be compromised; this literature review attempted to summarise this impact.

## 2. Method

Electronic databases including PubMed, PsycINFO, Google Scholar, PsycARTICLES, MEDLINE, Scopus, Wiley Online Library, and Proquest Psychology Journals were reviewed for studies published in peer-reviewed journals within the last decade. A large number of the identified studies were published in 2003, signalling an increase in focus on siblings around this time and a suitable start point for this historical investigation. Fourteen articles were accessed using key words to identify research within the sibling well-being field and further filtered in order to critically appraise the most relevant studies. Due to the small number of robust and empirically sound studies focussing specifically on siblings of children with autism, a number of studies focussing on the functioning of the whole family in addition to studies targeting a range of developmental disabilities were considered. Relevant studies were also identified if they were cited within the articles accessed through the above database search. Within the sibling field a number of pertinent concepts were identified; the current article will discuss the outcome of siblings of children with autism according to psychological health, overall quality of life, emotional intelligence, family coping, and child and family factors. 

The original criteria used to filter relevant articles allowed for inclusion of databased articles that were ASD-specific and included a control comparison group. However, these criteria needed to be relaxed as only 4 studies met this stringent category. No studies based exclusively on adolescent data met the criteria. This literature review revealed a need for further research into adolescent-specific outcome. 

## 3. Results

### 3.1. Psychosocial, Emotional, and Behavioural Outcome

*Pilowsky et al. [11].* Siblings of individuals with autism appear to be socially and emotional well adjusted according to a study that compared them to siblings of individuals with developmental language disorders (specific learning disability) and intellectual disability. Despite the absence of a typically developing sibling control comparison, these findings are still promising due to the low number of the siblings of individuals with ASD that fell into the clinical diagnostic range (13.4%) on a number of behavioural adjustment measures. 

*Kaminsky and Dewey [10]. *With the advantage of including comparison groups of siblings of individuals with another disability (Down Syndrome) and those with only typically developing siblings, Kaminsky and Dewey reported low levels of loneliness in siblings of individuals with ASD. They also reported that siblings of individuals with ASD were no more likely to have adjustment problems than comparison siblings. Despite all of the sibling groups falling into the “normal range” for adjustment, Kaminsky and Dewey concluded that it is more likely for siblings of individuals with ASD who live in larger families to be well-adjusted. The addition of other typically developing children in the family appeared to buffer the negative impacts of growing up with autism in the home. Many siblings reported high levels of social support, a promising finding that may have buffered against the adjustment problems reported in other studies. 

*Macks and Reeve [14]. *Children and adolescent's self-concepts appear to benefit from them having a sibling with ASD, when demographic risk factors are limited. The risk factors that are important for psychological well-being were not detailed in the study; however, birth order, family size, gender, and socioeconomic status (SES) were considered in their analyses. It is not clear which gender or birth order constitutes a risk for psychosocial and emotional maladjustment or whether a small family constituted a risk as reported previously; however, other studies reported below have investigated these variables. The important finding to note is that demographic factors may have a cumulative effect in producing poorer outcome. 

*Quintero and McIntyre [20]. *The social, behavioural, academic, and psychological adjustment of siblings was investigated in families with and without a child with ASD. Quintero and McIntyre found no significant differences between the groups. There was an interaction effect, however, between maternal well-being and sibling adjustment. Both teacher and parent reports suggested that higher levels of daily stress and depression in the mother correlated with higher ratings of problem behaviours in siblings. 

*Ross and Cuskelly [5]. *In a study that categorised 40 percent of siblings of individuals with autism in the borderline or clinical range for behavioural and emotional dysfunction, Ross and Cuskelly concluded that the risk of developing internalising behaviour problems is heightened in siblings of individuals with ASD. Aggression was identified as the most common stressor in the sibling relationship. 

*Orsmond and Seltzer [21]. *An interaction between the number of stressful life events experienced by siblings of individuals with ASD and their level of subthreshold ASD characteristics was used as evidence to partially support a diathesis-stress model of sibling adjustment. Genetic vulnerability to ASD (broad autism phenotype) was found to increase depressive and anxious traits only in the presence of high levels of stress, such as in maternal depression and challenging behaviours in the individual with ASD. The adolescent sisters in the sample reported more depression and anxiety symptoms than brothers but were comparable to community samples. The brothers reported considerably lower levels of anxiety and depression than the general population. It was reported that maternal depression was related to increased risk of depression in the adolescent siblings of individuals with ASD. 

*Verté et al. [13]. *Behaviour problems and social competence in siblings of individuals with high-functioning ASD were found to be comparable to a control group of siblings of typically developing individuals. Siblings appear to be capable of adapting to the environmental demands of high-functioning autism. 

*Hastings [22].* In comparison to a normative sample, siblings of individuals with ASD were rated higher on measures of behavioural dysfunction and exhibiting fewer prosocial behaviours. Brothers and younger siblings engaged in less prosocial behaviour within the target sample. Maternal stress and behaviour problems in the child with autism were not predictive of sibling adjustment; however, the low sample size reduces predictive power, a common difficulty in sibling research. The siblings with ASD were all recruited from one school, reducing demographic variability and lowering ability to generalise these results. It may be that the families receive similar opportunities for services and support. The study also failed to control for BAP (as does the majority of the sibling studies), so it is unclear whether these behaviour problems have genetic or environmental basis, particularly when the outcome measure selected is based on two important components of the triad of autism diagnosis (challenging behaviours and social dysfunction). These behavioural and social dysfunctions are potentially aspects of a genetic vulnerability to ASD. Hastings provides only a brief report and recognises the limitations of his data; however, his contribution is important, particularly as his analyses took into account important variables such as age, gender, gender matches, birth order, and living arrangements of siblings. 

*Benderix and Sivberg [16]. *A case study methodology revealed a number of stressful life conditions that siblings of individuals face regularly. A number of emotional reactions were identified within the seven domains of the sibling experience: empathy, sympathy, fear, anxiety; and social isolation. The siblings revealed that: (1) they felt obligated under a sense of precocious responsibility for protecting the individual with ASD and helping their parents; (2) they felt sorry for their sibling with ASD; (3) they were exposed to frightening and abnormal behaviour; (4) they held empathetic feelings for their sibling with ASD; (5) they hoped and wished that their family, and the individual with ASD may have some relief with the individual in a group home; (6) impulsive and uncontrolled physical violence made the siblings feel anxious and unsafe; and (7) their relationships with friends were negatively affected. Despite some positive emotions, the overwhelming feel of the sibling experience was negative. 

### 3.2. Quality of Life

*Moyson and Roeyers [8]. *In a recent qualitative investigation into the quality of life of siblings of children with ASD (SibQoL), Moyson and Roeyers [8] identified a number of integral domains within this concept. An important theme contributing to SibQoL appeared to be the “apparent invisibility of ASD” (page 46). A heterogeneous disorder, ASD does not always present uniformly and there are no physical features distinguishing individuals with ASD from their typically developing peers. Siblings report both negative and positive impacts of this “invisibility.” It may ensure that siblings do not have to explain autism to others. It may also ensure that others do not stare at the individual with ASD or express their negative opinions [8]. It does, however, lessen the likelihood that the outside world can accurately assess the impact of a child with ASD on their sibling/s, possibly lessening the likelihood that the sibling's feelings will be validated and that they will receive appropriate services. 

A further important domain of SibQoL was mutual understanding. The siblings felt it was important that the individual with ASD could communicate with them; they were disappointed that their sibling did not always want to talk, and they consistently felt misunderstood. 

Moyson and Roeyers [8] suggested that the characteristic behaviour of an individual with ASD, reported by their siblings as being sometimes “bizarre, aggressive, or annoying,” (page 47) could be hard to cope with. Siblings do appear to eventually develop awareness that the individual with ASD is often unable to control their behaviour, resulting in some forbearance on the part of the sibling. The qualitative findings of the study suggested that the siblings developed patience and tolerance to these challenging behaviours. Adjusting to these behaviours, however, may take time and support. Longitudinal studies would provide important information about the common trajectories across the lifespan for siblings. 

*Davis and Gavidia-Payne [23].* Quality of life in families of children with a disability was significantly accounted for by a number of family variables. These variables included: parental perceptions of the disability; experiences of family-centred professional support; perceived intensity of child behavioural problems; and support from extended family members. This has important implications for providing parents with the skills and opportunities to apply positive meaning to disability and model this to their family. It has been reported that parents use both positive and negative appraisals of childhood developmental disability [24]. These findings also suggest that disability services should focus on family approaches to intervention. 

### 3.3. Emotional Intelligence

Siblings of individuals with ASD have been reported to conceptualise the self in a positively enhanced manner in comparison to the general population [13]. Siblings of individuals with ASD were more likely to have developed positive perspectives towards their behaviour, intelligence, academic ability, and levels of anxiety. They also held a more positive view of their overall personal characteristics than siblings of typically developing individuals [14]. Furthermore, qualitative reports revealed empathetic feelings towards siblings with autism [16], showing an emotional maturity also reported by Gray [17]. 

### 3.4. Family Coping

*Bayat [12]. *A diagnosis of ASD often brings with it a period of family loss: feelings of loss for the individual with the disorder, parental ideas and plans for their family's future, and the loss of the “normal” sibling relationship for the typically developing child. Once the period of grieving is over, however, families often show great resilience in the face of adversity. Bayat identified a number of coping strategies used by families facing ASD that fit nicely with the foundational concepts of the resilience theoretical framework [25, 26]. Using qualitative and quantitative investigations, Bayat reported evidence of family unity, mobilisation of resources, greater life appreciation, and spiritual and personal growth. The families also appeared to attach positive meaning to the disability in many instances. These findings are encouraging for families. 

*Opperman and Alant [9]. *Qualitative data was used to identify adolescent perceptions and coping responses towards a sibling with a disability. Limited family interaction was found in families facing disability. Adolescent siblings resisted expressing feelings about the individual with the disability; however, they did express emotions related to guilt. Unfortunately this study was not specific to ASD, but it gives justification that qualitative studies should be pursued in the ASD field as the findings may have important implications for what components of their experience siblings feel comfortable in expressing. 

### 3.5. Family Factors and Demographic Variables

*Quintero and McIntyre [20]*. Maternal well-being correlated negatively with higher problem behaviours in siblings of individuals with ASD; however, there was no significant difference between siblings of individuals with ASD and those with typically developing siblings on measures of adjustment (behavioural outcome and socialisation skills). 

*Giallo and Gavidia-Payne [27]. *SES; past attendance at a sibling group; parent stress; family time and routines; family problem-solving and communication; and family hardiness were found to be important predictors of sibling adjustment in families of children with a disability. The study consisted of children with intellectual, sensory, physical, or developmental disabilities, with 30.6 percent of participating families including a child with ASD. 

*Orsmond et al. [28].* More positive effect has been reported within sibling relationships in which the member with ASD had fewer problem behaviours. 

## 4. Discussion

The present study aimed to determine the extent of negative and positive impacts of growing up with a sibling with ASD. 

 The scarce research available on psychosocial outcome points to some promising outcomes; the review revealed positive impacts of growing up with a sibling with ASD contingent upon limited risk factors. Only four studies met the more rigorous criteria of: quantitative study, focused exclusively on siblings of individuals with ASD with comparisons to controls with only typically developing siblings, and targeting behavioural and emotional psychosocial adjustment rather than the quality of the sibling relationship [10, 13, 14, 20]. All of these studies suggested that the sample siblings were well adjusted under optimal environmental conditions (limited demographic risk factors, large family, no compromised maternal well-being, sibling has high-functioning ASD). This suggests that the focal mechanism for poor outcome may not be the autism spectrum disorder but a number of other child and family variables. These studies, however, still involved a mixture of children and adolescents, or in the case of Quintero and McIntyre, primary school-aged children only; this creates a challenge in attempting to make conclusive remarks regarding adolescent sibling outcome and a complete lack of ability to comment on the adult experience. Furthermore, none of the databased studies explicitly measured QoL, and they all involved less than one hundred participants. 

The majority of studies involved children and adolescent samples, leading to confounding results and an inability to draw accurate insights and conclusions about the distinct life stages of childhood and adolescence. This is concerning due to the large changes that occur during transition through these life periods. Siblings play a vital role in each other's lives within the family system. During childhood, children rely heavily on siblings for a multitude of functions: as role models; in the development of theory of mind; in seeking personal identity and their niche in the family and then the world; and in developing socialisation skills. During this period siblings may fail to understand a complex disability like ASD and may not understand why their brother or sister will not play with them, why their brother or sister gets different rules, and why their parents spend more time with their brother or sister. 

During adolescence, teenagers begin to spend less time with siblings and family and more time with friends; they may begin to understand the diagnosis of ASD more and feel guilt for any of their previous behaviours toward their sibling. They have usually formed strong social awareness and may become embarrassed by disability in the family, potentially resulting in a conflict between loyalty to their sibling and a desire to fit in with their peers. With such different concerns, child and adolescent siblings need to be studied separately. Out of the two studies that focussed exclusively on an adolescent sample, one was not autism-specific and included multiple severe disabilities [9]. Limited family interaction and uncomfortable emotions were some concerning results that arose from this qualitative study. The other study concluded with a strong genetic environment interaction as a basis for the depressive and anxious traits found in the siblings of individuals with ASD [21], whereas the current literature review was more concerned with the psychosocial components of sibling outcome. 

 When QoL was investigated using qualitative measures, communication within the sibling relationship and the “invisibility of autism” emerged as important themes. The severity of communication deficits in the individual with ASD may be a mediating factor in QoL outcome studies and should be investigated in future research. 

 In regard to behavioural and emotional adjustment, internalising difficulties appear to be a concern for siblings of individuals with autism [5] but perhaps mostly in sisters [21] and in the presence of high levels of stress or maternal depression [20, 21].

 On a more positive note, Bayat [12] reported evidence of family unity, mobilisation of resources, greater life appreciation, and spiritual and personal growth. The families of individuals with ASD also appeared to attach positive meaning to the disability in many instances. Opperman and Alant [9] did find, however, that siblings of individuals with a disability felt that they could not express their feelings regarding the disability. This needs to be investigated in ASD-specific samples, with implications for service providers to give siblings the opportunity to express emotions such as guilt. Investigations into how we can foster resilience in our most at-risk families seem important. 

ASD may be conceptualised as a developmental disorder that is particularly disruptive to the family unit. This is supported by reports that siblings of individuals with intellectual disability without ASD were emotionally and behaviourally better adjusted than those siblings of individuals with intellectual disability and comorbid ASD [29]. This supports the notion that autism contributes to environmental stress that a sibling needs to adapt to, that it produces a unique set of circumstances for the sibling to navigate, and that autism specific studies, in the midst of such mixed results, need to become more prevalent in addition to disability studies. 

### 4.1. Implications

Despite reports that the large majority of siblings of individuals with ASD may be well adjusted, a significant proportion of the research indicates some vulnerability for behavioural and emotional dysfunction. This suggests that siblings need to be considered when planning interventions for ASD, as important units within whole family functioning. The current review helps to identify potentially at-risk siblings, such as those in small families and those with little external support. Furthermore, while sisters appear to internalise the stress of growing up with a sibling with ASD, brothers and younger siblings appear to be at heightened risk for externalising disorders and antisocial behaviours.

### 4.2. Future Directions

In summarising current knowledge of sibling outcome in ASD research and identifying the gaps in this knowledge, the current literature review provides a theoretical and empirical basis from which to develop a conceptual framework to test the important research questions that have yet to be answered. What impact does having a sibling with ASD have on children, adolescents, and adults as distinct life stages? What factors contribute to poor outcome in highly stratified sibling samples? Does poor outcome persist long term? What transitions are important in the life of the typically developing sibling and the individual with ASD?

Do sibling support programs result in better outcome after the program? Most importantly, *how can we lessen the impact of autism on siblings and families?*

With findings that suggest the true impacts of growing up with a sibling with a disability may be masked by samples containing too many confounding factors such as age and gender [14, 21, 30], further studies are vital in presenting conclusive findings. Studies using systematic sampling procedures to identify participants within a narrow age range and controlling for confounding variables such as gender and birth order need to become more common in the sibling literature. 

According to Hodapp et al. [30], research into the siblings of individuals with disabilities needs to meet certain vital criteria before the sibling community can reach a consensus. They identified six themes that appeared to be creating challenges in the sibling literature, providing a framework for guiding future research in this field. Methodological challenges, measurement inconsistencies, developmental and life-course perspectives, factors and predictors, cultural issues, and balanced views were revealed as potential challenges in presenting conclusive data. (1) Methodological challenges included the need to consider genetic and environmental influences, the use of systematic sampling procedures that take important characteristics into account (age, gender, and birth order), and the need for matched control groups. These difficulties present significant potential for confounding yet are common in the sibling literature to date. (2) Measurement difficulties result from the use of various assessments and methodologies. (3) A developmental perspective is yet to be arrived at due to the use of both children and adolescents within sibling samples. (4) Explicit research on mediators and moderators of sibling outcome specifically for ASD is scarce and has been identified by Hodapp et al. as an important consideration for future researchers. (5) Furthermore, there is a complete paucity of studies examining cultural factors in sibling research. (6) Lastly, Hodapp et al. valued the presentation of both positive and negative aspects of sibling adjustment. 

There is a need within the literature to further define well-being, a term that currently appears to encompass a large range of sibling outcome measures, as being distinct from adjustment and QoL, two potentially separate components of the well-being construct. It has been shown that psychosocial outcome and QoL are related, but distinct constructs and that psychosocial outcome may be a necessary but insufficient component of overall QoL [31]. A clearer distinction is needed of the terms used to represent the outcome measures used in future research. Despite normal levels of psychosocial adjustment in adolescents following childhood traumatic brain injury (TBI), participants reported low levels of QoL in comparison to healthy controls [31]. This suggests that QoL can still be affected in the presence of normal levels of psychosocial health. This justifies further investigation into QoL in disability studies, even if sibling adjustment appears normal, which is still debatable at this point. As it is yet to be explored empirically, it is only theoretically pertinent to propose at this point that siblings of individuals with ASD may be behaving well and reporting no ill effects of their home environment due to empathy for their sibling and a desire to assist their parents, despite implicitly suffering compromised satisfaction with life. 

The studies that reported that siblings of individuals with ASD were well adjusted included mostly demographically stable samples; hence, future research using demographically disadvantaged siblings is warranted. 

Many family and child factors have been linked to poorer outcome in siblings of individuals with a disability. These factors need to be considered in future studies so we can begin to make conclusive remarks on sibling outcome. These mediating variables do suggest, however, that there is the potential for siblings of individuals with ASD to become well adjusted and happy adults under the right conditions. These conditions need to be rigorously studied so we can optimise sibling well-being.

## 5. Conclusions

It appears that quantitative examples of the sibling experience, in which researchers categorise siblings into clinical and nonclinical or rate their symptom level in the desired emotional and behavioural domains, show that siblings may be well adjusted if not for a small proportion of vulnerable children. Qualitative data, however, reveals some disturbing emotional and cognitive challenges for siblings of individuals with ASD, justifying further investigation into subjective well-being outcomes. These siblings may be behaving and coping well, while internally facing turmoil. 

## References

[1] Stone WL, McMahon CR, Yoder PJ, Walden TA (2007). Early social-communicative and cognitive development of younger siblings of children with autism spectrum disorders. *Archives of Pediatrics and Adolescent Medicine*.

[2] Warren ZE, Foss-Feig JH, Malesa EE ( 2012). Neurocognitive and behavioral outcomes of younger siblings of children with autism spectrum disorder at age five. *Journal of Autism and Developmental Disorders*.

[3] American Psychiatric Association (2000). *Diagnostic and Statistical Manual of Mental Disorders*.

[4] Bartak L (2011). *Having Another Diagnosis With Autism Spectrum Disorder*.

[5] Ross P, Cuskelly M (2006). Adjustment, sibling problems and coping strategies of brothers and sisters of children with autistic spectrum disorder. *Journal of Intellectual & Developmental Disability*.

[6] Rodrigue JR, Geffken GR, Morgan SB (1993). Perceived competence and behavioral adjustment of siblings of children with autism. *Journal of Autism and Developmental Disorders*.

[7] Orsmond GI, Seltzer MM (2007). Siblings of individuals with autism spectrum disorders across the life course. *Mental Retardation and Developmental Disabilities Research Reviews*.

[8] Moyson T, Roeyers H (2011). The quality of life of siblings of children with autism spectrum disorder. *Exceptional Children*.

[9] Opperman S, Alant E (2003). The coping responses of the adolescent siblings of children with severe disabilities. *Disability & Rehabilitation*.

[10] Kaminsky L, Dewey D (2002). Psychosocial adjustment in siblings of children with autism. *Journal of Child Psychology and Psychiatry and Allied Disciplines*.

[11] Pilowsky T, Yirmiya N, Doppelt O, Gross-Tsur V, Shalev RS (2004). Social and emotional adjustment of siblings of children with autism. *Journal of Child Psychology and Psychiatry and Allied Disciplines*.

[12] Bayat M (2007). Evidence of resilience in families of children with autism. *Journal of Intellectual Disability Research*.

[13] Verté S, Roeyers H, Buysse A (2003). Behavioural problems, social competence and self-concept in siblings of children with autism. *Child*.

[14] Macks RJ, Reeve RE (2007). The adjustment of non-disabled siblings of children with autism. *Journal of Autism & Developmental Disorders*.

[15] Rivers JW, Stoneman Z (2003). Sibling relationships when a child has autism: marital stress and support coping. *Journal of Autism & Developmental Disorders*.

[16] Benderix Y, Sivberg B (2007). Siblings’ experiences of having a brother or sister with autism and mental retardation: a case study of 14 siblings from five families. *Journal of Pediatric Nursing*.

[17] Gray DE (1998). *Autism and the Family: Problems, Prospects, and Coping With the Disorder*.

[18] Smith L, Elder J (2010). Siblings and family environments of persons with autism spectrum disorder: a review of the literature. *Journal of Child and Adolescent Psychiatric Nursing*.

[19] Meadan H, Stoner JB, Angell ME (2010). Review of literature related to the social, emotional, and behavioral adjustment of siblings of individuals with autism spectrum disorder. *Journal of Developmental and Physical Disabilities*.

[20] Quintero N, McIntyre LL (2010). Sibling adjustment and maternal well-being: an examination of families with and without a child with an autism spectrum disorder. *Focus on Autism and Other Developmental Disabilities*.

[21] Orsmond GI, Seltzer MM (2009). Adolescent siblings of individuals with an autism spectrum disorder: testing a diathesis-stress model of sibling well-being. *Journal of Autism and Developmental Disorders*.

[22] Hastings RP (2003). Brief report: behavioral adjustment of siblings of children with autism. *Journal of Autism and Developmental Disorders*.

[23] Davis K, Gavidia-Payne S (2009). The impact of child, family, and professional support characteristics on the quality of life in families of young children with disabilities. *Journal of Intellectual and Developmental Disability*.

[24] Trute B, Hiebert-Murphy D, Levine K (2007). Parental appraisal of the family impact of childhood developmental disability: times of sadness and times of joy. *Journal of Intellectual & Developmental Disability*.

[25] Walsh F (1996). The concept of family resilience: crisis and challenge. *Family Process*.

[26] Walsh F (1998). *Strengthening Family Resilience New York*.

[27] Giallo R, Gavidia-Payne S (2006). Child, parent and family factors as predictors of adjustment for siblings of children with a disability. *Journal of Intellectual Disability Research*.

[28] Orsmond GI, Kuo H-Y, Seltzer MM (2009). Siblings of individuals with an autism spectrum disorder: sibling relationships and wellbeing in adolescence and adulthood. *Autism*.

[29] Petalas MA, Hastings RP, Nash S, Lloyd T, Dowey A (2009). Emotional and behavioural adjustment in siblings of children with intellectual disability with and without autism. *Autism*.

[30] Hodapp RM, Glidden LM, Kaiser AP (2005). Siblings of persons with disabilities: toward a research agenda. *Mental Retardation*.

[31] Green L, Godfrey C, Soo C, Anderson V, Catroppa C A preliminary investigation into psychosocial outcome and quality of life in adolescents following childhood traumatic brain injury.

